# Climate and Soil Characteristics Determine Where No-Till Management Can Store Carbon in Soils and Mitigate Greenhouse Gas Emissions

**DOI:** 10.1038/s41598-019-47861-7

**Published:** 2019-08-12

**Authors:** Stephen M. Ogle, Cody Alsaker, Jeff Baldock, Martial Bernoux, F. Jay Breidt, Brian McConkey, Kristiina Regina, Gabriel G. Vazquez-Amabile

**Affiliations:** 1 Natural Resource Ecology Laboratory, Colorado State University, Fort Collins, Colorado, 80523 USA; 2 Department of Ecosystem Science and Sustainability, Colorado State University, Fort Collins, Colorado, 80523 USA; 3 CSIRO Agriculture and Food, Locked Bag 2, Glen Osmond, SA 5064 Australia; 4 Food and Agriculture Organization of the United Nations (FAO), Climate and Environment Division, Rome, Italy; 5 Department of Statistics, Colorado State University, Fort Collins, Colorado, 80523 USA; 6 Agriculture and Agri-Food Canada, Swift Current, SK S9H3X2 Canada; 7 Natural Resources Institute Finland, FI-31600 Jokioinen, Finland; 8 School of Agronomy and Forest Engineering, National University of La Plata, Diag 113-N° 469, (1900) La Plata, Argentina

**Keywords:** Agroecology, Carbon cycle

## Abstract

Adoption of no-till management on croplands has become a controversial approach for storing carbon in soil due to conflicting findings. Yet, no-till is still promoted as a management practice to stabilize the global climate system from additional change due to anthropogenic greenhouse gas emissions, including the 4 per mille initiative promoted through the UN Framework Convention on Climate Change. We evaluated the body of literature surrounding this practice, and found that SOC storage can be higher under no-till management in some soil types and climatic conditions even with redistribution of SOC, and contribute to reducing net greenhouse gas emissions. However, uncertainties tend to be large, which may make this approach less attractive as a contributor to stabilize the climate system compared to other options. Consequently, no-till may be better viewed as a method for reducing soil erosion, adapting to climate change, and ensuring food security, while any increase in SOC storage is a co-benefit for society in terms of reducing greenhouse gas emissions.

## Introduction

Over two decades ago, scientists began proposing no-till management as a way to mitigate greenhouse gas (GHG) emissions through carbon (C) storage in soils^1–3^. No-till, as the name implies, is the practice of direct-seeding of crops in a field without ploughing. The initial rationale for developing and adopting no-till management during the latter half of the 20^th^ Century was to reduce soil degradation from erosion, and no-till has proven highly effective for achieving this goal^4–6^. No-till can also improve nutrient cycling^7^, and in semi-arid and arid regions where water limits crop production, no-till can conserve water in the soil by improving infiltration and reducing evaporation with a sufficient cover of crop residues or cover crops^8–10^. Therefore, no-till management has been promoted as a win-win situation for climate change policy by addressing GHG mitigation goals and enhance resilience to meet adaptation goals.

Scientists had a seemingly strong basis for promoting adoption of no-till management in croplands as part of a group of options to reduce the concentration of GHGs in the atmosphere and stabilize the climate system^11^. First, it was well known that conversion of forests and grasslands to croplands, which are cultivated by ploughing, leads to a large decline in soil organic C (SOC)^12^. Second, research demonstrated that no-till enhanced macroaggregate stability and microaggregate formation that led to greater protection of C from microbial decomposition^13,14^. Third, meta-analyses provided empirical evidence that there was more C in soils with conversion from an intensive, full tillage practice to no-till management^15,16^. Consequently, both empirical and mechanistic studies appeared to confirm that no-till led to higher amounts of C in soils, and over the past two decades, no-till has generally been a key part of GHG mitigation analyses^17^.

However, scientists began asking questions about the effectiveness of no-till as a way to mitigate GHG emissions^18–20^. After all, not all studies found that SOC stocks increased with no-till management^21^. Studies analyzed from Eastern Canada showed that inversion tillage incorporates crop residue C deeper into the soil profile, slowing decomposition and negating some of the benefit of no-till for storing C^22^. This led investigators to the conclusion that no-till management tends to increase C in the surface layer, but full tillage increases SOC deeper in the profile^23^. In addition, no-till tends to compact the topsoil, which increases the mass of the soil in the upper layer, compared to a soil that is ploughed. In fact, studies showed that there is no difference in the amount of C in soils with adoption of no-till management compared to full tillage, and that differences in SOC from the surface to the subsoil below the plow layer are explained by compaction and redistribution of SOC^24^. However, it has also been recognized that limited data have been collected at deeper depths, and that more samples are required to determine if no-till has an influence on the amount of SOC in the subsoil due to greater inherent variability^25,26^. These studies highlight the critical need for measuring SOC in the subsoil to determine the potential for no-till to sequester C^26^.

A variety of implements can be used to prepare soils for planting crops that lead to different levels of soil disturbance and incorporate C in plant residue litter from the surface to different depths. Full tillage with a traditional moldboard plough involves inversion of the soil, but tillage can also be done with implements that mix the soil without inversion, such as chisels, sweeps, and/or discs. In a global meta-analysis, investigators^27^ found that mixing tillage led to larger SOC stocks to 15 cm compared to inversion tillage. However there was also a trend of less SOC below 15 cm for non-inversion tillage such that there was no apparent difference in SOC stocks between mixing and inversion tillage. In addition, some studies have found no differences between the amount of SOC in surface soils managed with mixing tillage implements and no-till^28,29^, although other studies have found more C in soils managed with no-till^30^. Regardless, mixing tillage is frequently shallower than inversion tillage and the potential increased C retention with mixing tillage has been attributed to less soil disturbance compared to deeper inversion tillage management^31^. In addition to redistribution of organic C in the soil profile, crop production and the input of organic C to soils may decline with no-till management, especially in cooler and wetter climates, suggesting that SOC stocks may be reduced with conversion from full tillage to no-till^32,33^. However, crop production may not change or may even increase with no-till; for example, production is enhanced in dry climates if no-till is adopted in combination with residue retention in fields and planting of cover crops^33^.

No-till has become a controversial contribution in a portfolio of options for stabilizing the climate. Regardless, no-till is still a prominent part of GHG mitigation discussions, such as the 4 per mille initiative that has an ambitious goal of increasing global SOC stocks by 0.4% per year^34^. Therefore, we have compiled a comprehensive global dataset of 178 experimental sites with 1205 observations of changes between full tillage and no-till management to further evaluate this practice and determine if it is a viable mitigation option (See Supplementary Table S1). Full tillage typically refers to soil management with a farming implement that inverts or mixes soil to 15 cm or deeper, and leaves less than 30% of the soil’s surface covered with leftover crop residues or litter. No-till is direct seeding of cropland without preparing the soil with a tillage practice, and typically has more than 30% cover of the soil surface with residues. However, these criteria vary regionally depending on the climate and productivity of the system. For example, crop production in rain fed systems of semi-arid regions does not always produce enough residues to cover 30% of the soil surface due to low productivity. This would lead to classification as full tillage based on the general criteria even if no-tillage was practiced. To address these discrepancies, we accepted local classifications of full tillage and no-till as designated by the investigators in their publications even if they differed from the general criteria.

Our objective is to address the influence of no-till adoption on C storage potential of soils across a range of climatic conditions and soil types. With a global dataset, we have evaluated differences among cool temperate, warm temperate and tropical climates with moist/wet and dry moisture regimes, as well as sandy versus loamy, silty and clayey textured soils. If policy programs are to encourage no-till adoption, it is critical to know the conditions in which this practice can enhance SOC stocks. In addition, this analysis is unique from other studies in that we have analyzed the impact of no-till on SOC stocks as a continuous function of depth^35^. This allowed us to use all of the available studies and model SOC without aggregating data to specified depth increments or eliminating studies that did not measure soil C to a specified depth.

## Results

**Figure 1:**
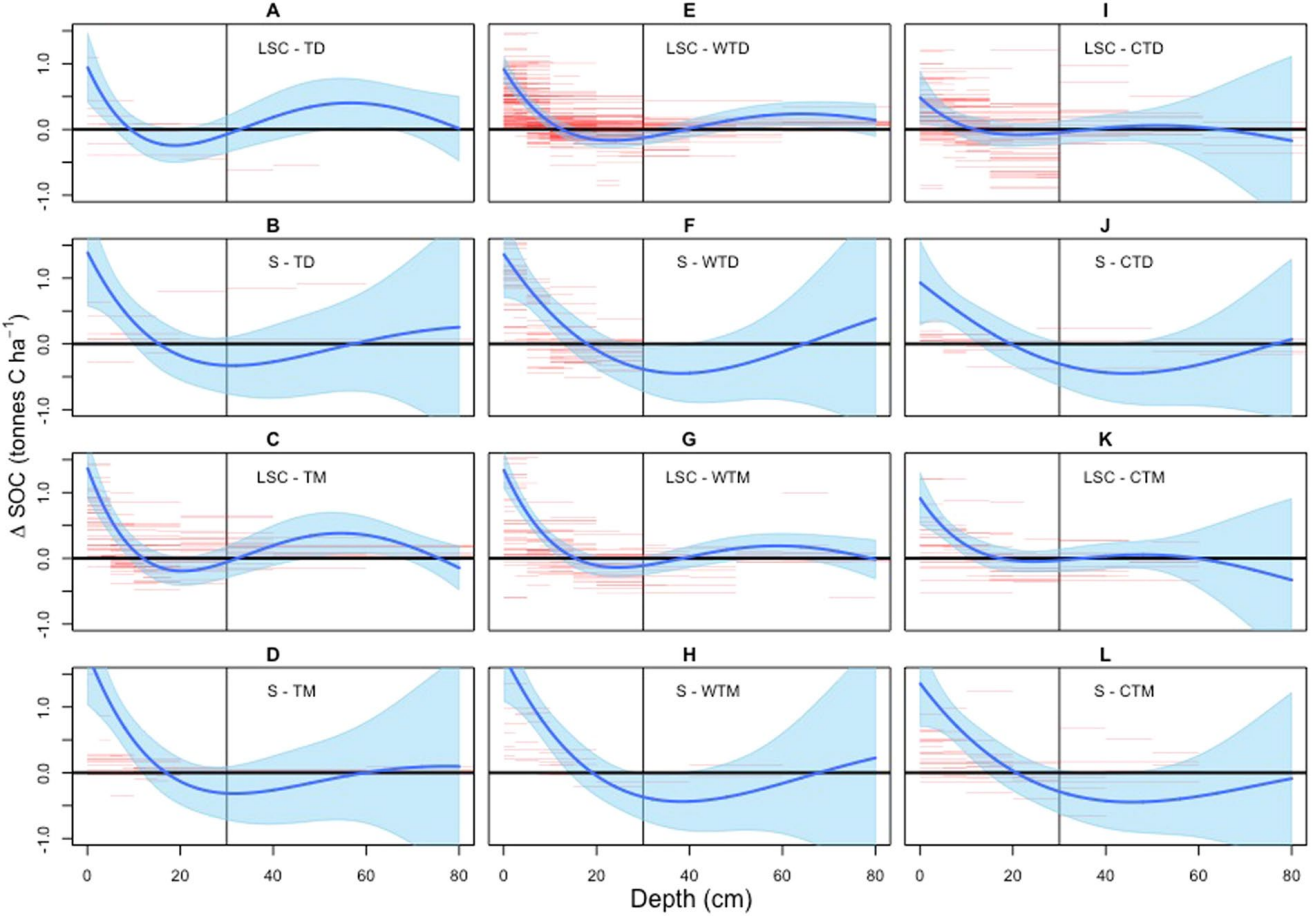
Change in soil organic C (tonnes C ha^−1^) between no-till and full tillage management across depths from 0 to 80 cm over 20 years. Positive values represent more soil organic C with no-till management. Graphs include the following climatic and edaphic conditions: (**A**) loamy, silty and clayey (LSC) soils in tropical dry climates, (**B**) sandy soils in tropical dry climates, (**C**) LSC soils in tropical moist/wet climates, (**D**) sandy soils in tropical moist/wet climates, (**E**) LSC soils in warm temperate dry climates, (**F**) sandy soils in warm temperate dry climates, (**G**) LSC soils in warm temperate moist climates, (**H**) sandy soils in warm temperate moist climates, (**I**) LSC soils in cool temperate dry climates, (**J**) sandy soils in cool temperate dry climates, (**K**) LSC soils in cool temperate moist climates, and (**L**) sandy soils in cool temperate moist climates. Sandy soils contain 50% or more sand. The red lines represent the observed changes in soil organic C at the depth range of each line, the blue shading is a 95% confidence interval for the estimated change in SOC stocks, and grey vertical line denotes a depth of 30 cm. These results are based on the measured differences at each depth without adjustment for equivalent mass (See Table 1 for adjusted results).

We first evaluated differences in SOC stocks (ΔSOC) at specific depths in the soil profile. The ΔSOC varied by depth between full tillage and no-till management, consistent with previous studies^23,24^. Two trends were evident based on the modelled responses of ΔSOC with soil depth: a) no-till had higher SOC stocks in the surface topsoil at depths less than 20 cm and b) soils with full tillage management had higher SOC at depths greater than 20 cm, which extended below the typical plough layer depth of 20–25 cm (Fig. 1).

There were differences among climate and soils types. The impact of tillage extended deeper in the profile for loamy, silty and clayey soils in tropical and warm temperate climates, compared to sandy soils in those climates. For example, the confidence interval for ΔSOC did not include 0 at depths around 60 cm for warm temperate dry climates (Fig. 1E, Blue Shading), while sandy soils did not have a significant impact on ΔSOC at depths below 35 cm for warm temperate dry climates (Fig. 1F, Blue Shading). This trend was reversed in cool temperate climates with significant impacts on ΔSOC around 20 cm for loamy, silty and clayey soils (Fig. 1I,K), but extending to depths around 40 cm for sandy soils (Fig. 1J,L).

Beyond 60 cm, limited data were available to assess differences in SOC storage for any of the soil types and climatic conditions (Fig. 1), and so it was not feasible to statistically evaluate the impact of no-till management deeper in the soil profile^25^. In addition, there was generally less data coverage across depths for sandy soils regardless of climate conditions and edaphic characteristics, and consequently larger uncertainties existed in estimated impacts of no-till management compared to the results for loamy, silty and clayey soils. In terms of climate, there were less data for tropical climates compared to temperate climates with the exception of loamy, silty, and clayey soils in wet/moist conditions.

One mechanism contributing to changes in ΔSOC with depth is that full tillage reduces bulk density and essentially raises the soil surface and increases the depth of the soil profile^36^. This leads to a larger mass of soil in the surface topsoil with no-till management, and therefore a higher SOC stock when calculated on a volumetric basis. We evaluated the change in soil mass with depth based on the studies that provided enough information to calculate the mass (See Supplementary Information). Soils with loamy, silty and clayey textures under no-till management did have greater mass in the surface topsoil and less mass below the topsoil based on a statistical analysis of studies providing data on soil mass (Figure S1 in Supplementary Information). There was no evidence of differences in mass for the surface and subsurface layers of sandy textured soils. Therefore, SOC stock estimates for loamy, silty and clayey textured soils were adjusted for mass equivalency between full tillage and no-till management. Mass equivalency adjustments ensure that the observed changes in SOC stocks are due to enhanced levels of organic C in the soil from processes such as protection of organic matter in microaggregates, and not due to sampling a different mass of soil.

In the second part of the analysis, we predicted ΔSOC stock changes with conversion of full tillage management to no-till for each of the climate and soil types. The predictions are based on the cumulative change to a minimum depth of 30 cm, or to a deeper depth if there are significant ΔSOC at a deeper depth based on the first part of the analysis. For the first 20 years following conversion from full tillage to no-till, the change in SOC stocks is predicted to be greatest in loamy, silty and clayey soils of tropical moist/wet climates at 0.54 tonnes C ha^−1^ yr^−1^, with a 95% confidence interval from 0.04 to 1.02 tonnes C ha^−1^ yr^−1^ (Table 1). Sandy soils in warm temperate moist climates also have relatively large changes in SOC predicted to be 0.5 tonnes C ha^−1^ yr^−1^, with a 95% confidence interval from 0.24 to 0.76 tonnes C ha^−1^ yr^−1^. Higher amounts of SOC, between 0.27 and 0.39 tonnes C ha^−1^ yr^−1^, are also predicted for sandy soils in cool temperate moist and tropical climates, in addition to loamy, silty and clayey soils in cool and warm temperate moist climates. Predictions for all remaining combinations of soil types and climates have estimated ΔSOC that are positive, but the confidence intervals for these climates and soil types include values that are less than 0. This includes all soil types in cool and warm temperate dry climates, and loamy, silty, and clayey soils in tropical dry climates.

**Table 1:** Change in SOC stock (tonnes C ha^−1^ yr^−1^) and 95% confidence intervals (in parentheses) over 20 years for different climates and edaphic (i.e., soil texture) conditions.

Temperature Regime	Moisture Regime	Soil Texture	Depth with Significant Differences in SOC (cm)	Delta SOC (tonnes C ha^−1^ yr^−1^)
Cool	Dry	Loamy, Silty, and Clayey	5	0.06 (−0.08, 0.18)
Cool	Dry	Sandy	40	0.15 (−0.15, 0.44)
Cool	Moist	Loamy, Silty, and Clayey	10	0.27 (0.00, 0.56)
Cool	Moist	Sandy	40	0.35 (0.05, 0.65)
Warm	Dry	Loamy, Silty, and Clayey	70	0.21 (−0.15, 0.52)
Warm	Dry	Sandy	40	0.18 (−0.10, 0.46)
Warm	Moist	Loamy, Silty, and Clayey	60	0.33 (0.03, 0.61)
Warm	Moist	Sandy	35	0.50 (0.24, 0.76)
Tropical	Dry	Loamy, Silty, and Clayey	65	0.34 (−0.19, 0.85)
Tropical	Dry	Sandy	5	0.39 (0.18, 0.60)
Tropical	Moist/Wet	Loamy, Silty, and Clayey	65	0.54 (0.04, 1.02)
Tropical	Moist/Wet	Sandy	10	0.35 (0.13, 0.55)

The depth with significant differences in SOC stocks is the deepest depth in the profile with a change in SOC stock that does not include 0 (See Figure 1 for differences in SOC between management practices from 0 to 80 cm). The Delta SOC estimate is based on a minimum depth of 30 cm, but includes differences in SOC to deeper depths if the depth with significant differences was greater than 30 cm (See Figure 1 for differences in SOC between management practices at depths from 0 to 80 cm). Loamy, silty and clayey soils were adjusted for mass equivalency.

## Discussion

Adopting no-till management has many benefits for sustainable management of soils, such as improving soil structure, reducing erosion, enhancing soil moisture, and C storage^5^. While many articles have promoted the last of these benefits^37^, our results suggests that no-till adoption on existing cropland is not universally applicable for mitigation of greenhouse gas emissions. In particular, reduced amounts of SOC deeper in soils may offset an increased amount of SOC near the soil surface with no-till management^22–24,38^. However, there is a sufficient amount of SOC in the topsoil according to our analysis to enhance the amount of SOC across the profile in sandy soils of tropical moist/wet, tropical dry, warm temperate moist and cool temperate moist climates, as well as loamy, silty and clayey soils in tropical moist/wet, warm and cool temperate moist climates. The results are less conclusive for all soil types in cool and warm temperate dry climates, and loamy, silty, and clayey soils in tropical dry climates. Our mean estimates imply that ΔSOC is higher in soils managed with no-till, but there is also a chance that ΔSOC may be negative according to our confidence intervals (Table 1). Therefore, we cannot conclude that soils managed with no-till have more SOC than soils managed with full tillage for these soil types and climates.

It is important to note that in some cases, the larger amount of SOC in no-till soil may represent an increase or sequestration of C from the atmosphere if the soil is gaining C overall, but in other cases, may represent a lower loss of C, for example if a native grassland or forest land has been converted to cultivated cropland during recent years^39^. In either case, there is more C in the soil with no-till management compared to full tillage management, and therefore less C in the atmosphere.

In general, ΔSOC is greater in warmer and wetter climates than drier and cooler climates^16^. The reasons for this cannot be determined based on our data, but explanations are likely related to differences in C input, decomposition rates and physical protection of C in the soil. In general, no-till reduces soil disturbance and increases aggregate stability, and enhances SOC in surface soils^14^. However, there are constraints on physical protection of SOC in colder environments due to soil freezing and thawing that disrupts soil aggregates^40^, and in drier environments due to rewetting of soils that can accentuate SOC mineralization^41^. Such processes may reduce the positive effect of no-till on C storage in soils that occur in drier and cooler climates. Another possibility is that crop production and C input is reduced with no-till management in drier and cooler climates. The literature on productivity differences between tilled and no-till soils does not indicate productivity and C input increases with no-till adoption in moist or wet climates^42^ and some results even suggest that there could be declines in productivity, particularly in cooler and wetter conditions^32,33^. Furthermore, no-till has been shown to increase crop productivity under drier conditions, which may be explained by the benefit of no-till for enhancing residue cover and soil moisture availability^43,44^. Soil biota also may react differently to management changes in dry compared to moist conditions. For example soil fauna may increase more in drier climates compared to wet climates following no-till adoption, thus increasing decomposition of residues^45^. However, soil fauna may also stabilize SOC in soil aggregates^40^, and therefore, it is not clear if the effect of soil faunal activity leads to a decrease or increase in SOC stocks^46–48^.

There was a lack of detectable differences between inversion and mixing tillage. This suggests that turning the soil with inversion tillage and placing residue at a deeper depth compared to mixing in residues throughout the tilled soil layer does not lead to more SOC deeper in the profile with full tillage compared to no-till management. This is similar to other studies that have also failed to detect differences between these tillage types^27–29,49–51^. Furthermore, there have been studies that found significantly higher SOC stocks with no-till adoption compared to systems with tillage management, but no differences between inversion tillage and reduced tillage management that also mix residue into the topsoil without inversion^28^. Additional studies, particularly those that include both tillage types in the same experiment and measure SOC stocks deep into the profile are needed to further evaluate differences in stocks over the soil profile between inversion and mixing tillage. Until the differences between mixing and inversion tillage are determined with confidence, it is appropriate to pool these full tillage types to quantify the effect of no-till adoption on SOC stocks.

Erosion and deposition are key processes influencing the amount of SOC storage across landscapes. Eroded sites tend to have greater potential to stabilize organic C on soil mineral surfaces that are brought into the topsoil with erosion of soil on the surface; and deposition sites tend to bury C deeper in the profile where decomposition rates are lower^52,53^. In fact, investigators have concluded from a global analysis that these processes have increased SOC storage by 78 Pg C in the terrestrial biosphere due to cultivation^54^. Agricultural experiments, where our data mainly originates, tend to be conducted on level terrain in which erosion and deposition are limited, and so our estimated ΔSOC does not likely incorporate this impact. Adopting no-till in fields subject to high erosion will reduce erosion due to protection of soil surface by crop residues. Retaining the topsoil likely better maintains productivity and thereby C input to the soil compared to sites allowed to become severely eroded over time to the point that production significantly declines. Maintaining productivity benefits food security and the associated C input will increase the *in situ* C levels in soils. While studies suggest that tilling soils and enhancing erosion may increase C storage in soils^54^, such practices cannot be recommended on highly erodible sites as a way to contribute to climate stabilization due to loss of substrate for crop production. The adoption of no-till on erodible sites may still be appropriate for food security as well as to reduce other negative effects of erosion regardless of the net effect on SOC storage across landscapes.

Our results have large uncertainties with confidence intervals that include rates of less than 0.1 tonnes C ha^−1^ yr^−1^ for many of the regions. In particular, uncertainty is high for loamy, silty and clayey soils, which is largely due to the equivalent mass adjustment associated with the impact of tillage on soil bulk density^36^. Sandy soils have a less developed structure, and so tillage appears to have limited or at least an ephemeral impact on soil bulk density, and consequently less uncertainty based on our analysis.

It is also important to recognize that longer-term impacts of no-till management on SOC storage cannot be quantified from the existing experimental data. The longest study in our dataset is 45 years (See Supplementary Material, Table S1). Long-term stabilization of C in soils is linked with bonding of microbial products in organo-mineral complexes^55,56^. It is conceivable that the reduction of C in subsoils of no-till management systems could be reversed over time as microbial products in dissolved organic matter are leached from the topsoil into the subsoil and stabilized in mineral-associated organic matter. Further study will be needed to understand the longer-term dynamics associated with no-till adoption.

It is essential to recognize that C storage has not been nor will be the primary reason to adopt no-till although this could be a misconception from articles that focus on this issue^37^. The value of no-till in minimizing erosion on cropland is well established worldwide to prevent or reverse soil degradation^4,5,57–60^. Furthermore, no-till adoption increases water conservation and crop water use efficiency, and investigators have concluded that global adoption of conservation agriculture, including no-till, is needed to overcome inherent water-related limitations to crop production if the world is to meet future food requirements^61^. Greater water-use efficiency is related to direct benefits of crop residue left on the surface in reducing loss of water from evaporation^62^ as well as improved infiltration and soil water-holding capacity^63^. No-till can also improve resource use efficiency through enhanced nutrient retention and availability. In a global meta-analysis, investigators^64^ found no-till increased the amount of N mineralization by 13% compared to full tillage, and no-till can enhance cation exchange capacity^65^, which increases retention of several plant nutrients, such as potassium. No-till can also reduce the number of field operations and days that are needed to plant crops^66^, which in turn, reduces fuel usage and associated GHG emissions^67,68^.

Even though at least 178 experiments have been conducted evaluating the impact of no-till on SOC, there is still insufficient information to make precise estimates of the ΔSOC, particularly deeper in the soil profile^25^. Some combination of process-based model development and soil monitoring networks may lead to more precise predictions of ΔSOC potential in the future^69,70^, but it seems unlikely that there will be sufficient studies in the near term to significantly reduce these uncertainties. Therefore, policy programs will need to consider the risk associated with the uncertainty in ΔSOC from no-till adoption relative to other options for stabilizing the climate by reducing anthropogenic greenhouse gas emissions. Other benefits of no-till adoption may be provide more compelling reasons for promoting this management practice^71^, with C storage being a co-benefit for society to the extent that greenhouse gas emissions are reduced.

## Methods

A literature review was conducted for studies meeting the following criteria. First, studies needed to be experiments with a control and treatment(s), and include measurements of SOC stocks or the data needed to compute stocks (bulk density, organic C concentration, and gravel content). Second, we required the depth of the measurements and number of years from the beginning of the experiment. Furthermore, short-term studies and studies that only sampled the topsoil were not excluded as has been done in other meta-analyses^38^ because we applied a regression modeling method to predict changes in SOC as a function of time since no-till adoption and as a function of depth without aggregation. Third, soil texture data were needed to evaluate the impact of edaphic conditions and location of the experiment to assess the impact of climate. Fourth, we required sufficient information to classify full tillage into mixing and inversion practices. We did not exclude studies because there was not enough information to estimate equivalent mass. However, we did extract data when available to estimate the change in mass, and address mass equivalency^23,24,38^. We compiled data from 178 experimental sites with 1205 observations based on these criteria (See Supplementary Table S1).

A semi-parametric mixed effect models was developed to estimate the difference in SOC between no-till and full tillage management^35^. The response variable (Δ*SOC*) is

1 $${\rm{\Delta }}SOC=SO{C}_{NT}-SO{C}_{FT},$$

where SOC stock under full tillage (*SOC*_*FT*_) is subtracted from the SOC stock under no-till management (*SOC*_*NT*_). Several predictor variables were tested for the model including depth; number of years since the management change; climate; soil texture classes for sandy (greater than 55% sand) compared to loamy, silty and clayey soils; the type of full tillage, i.e., inversion and mixing tillage; in addition to two-way interactions among the variables. Variables were retained in the model if they met an alpha level of 0.05 and decreased the Akaike Information Criterion by two.

Data were not aggregated to a standardized set of depths but rather all of the original depth increments as measured in the experiments were used in the analysis as separate observations of SOC stock changes (e.g., 0–5 cm, 5–10 cm, and 10–30 cm). Similarly, time series data were not aggregated, even though those measurements are taken from the same plots. Consequently, random effects were included to account for the dependencies in times series data and among data points representing different depths from the same experimental site.

Data were collected by researchers at various depths that did not match among studies, and measurements correspond to depth increments (e.g., 10–30 cm) rather than a specific depth (e.g., 14 cm). Such increment data require special consideration for statistically valid inference^35^. Depth appears as an explanatory variable in the model not directly, but as a custom covariate obtained as the definite integral of depth over each increment in each study. For example, the integral of depth is (depth)^2^/2, therefore the value of the custom covariate for 10–30 cm is 30^2^/2 – 10^2^/2 = 400. Similarly, each function of depth (e.g., quadratic or other polynomial functions of depth to capture curvature associated with SOC differences between tillage practices) that is used as an explanatory variable required its own custom covariate, again obtained as the definite integral of the function over each increment. Additionally, these custom depth covariates were interacted with years and all other variables. This approach was needed to make statistically valid inferences with the semi-parametric mixed effect model^35^.

The best-fit statistical model contained all the variables except the type of full tillage practice (inversion versus mixing), which was not significant at the 0.05 alpha level. The model also included the two-way interactions between the number of years since the management change and each of the other individual variables.

We developed and applied a second model to modify the predictions by adjusting for mass-equivalency^36^. We used a modified approach compared to other studies^24^ in which we adjusted the predicted Δ*SOC* estimates from the semi-parametric mixed-effect model rather than the initial dataset prior to the analysis. This approach allowed us to include all observations from the experiments in the development of the Δ*SOC* estimates, rather than losing information by excluding studies that did not provide sufficient data to estimate mass-equivalency for the depth increments.

The model predicting the difference in soil mass between full tillage and no-till management was based on 33 experimental sites with 214 observations (See Supplementary Table S1). We used the same semi-parametric modeling approach described above and tested the same covariates for significance. The final model included depth and the number of years since the management change. Sandy soils did not have statistically significant differences in soil mass between full tillage and no-till management so data for this soil type were removed from the analysis, and so the Δ*SOC* estimates for sandy soils were not adjusted for mass equivalency (More information about the model is provided in the Supplementary Information).

Estimates and uncertainty in Δ*SOC* were derived using a Monte Carlo simulation approach to produce a mean and 95% confidence interval for each result. For sandy soils, the distribution of Δ*SOC* was approximated with 1000 random draws of parameter values from the joint probability distributions of the fixed effects in the semi-parametric mixed effect model predicting ΔSOC (Equation 1). For loamy, silty and clayey soils, a distribution of ΔSOC was produced using the same procedure as sandy soils (Equation 1), but these values were adjusted for mass equivalency (Supplementary Equation S2) based on 1000 random draws of values for the fixed effects in the semi-parametric mixed effect model predicting the change in soil mass (Supplementary Equation S1).

## Supplementary information

Supplementary Information

## Data Availability

The data used in the analysis of soil organic C stock changes are provided in Supplementary Information.
