# Supplementary material for: Climate and Soil Characteristics Determine Where No-Till Management Can Store Carbon in Soils and Mitigate Greenhouse Gas Emissions
Source: Sci Rep. 2019 Aug 12;9:11665. doi: 10.1038/s41598-019-47861-7 (PMC6691111; doi:10.1038/s41598-019-47861-7)
Supplement: Supplementary file 1 — Supplementary Information [file 41598_2019_47861_MOESM1_ESM.pdf]

Supplementary Information

Climate and Soil Characteristics Determine Where No-Till Management Can Store Carbon in Soils and Mitigate Greenhouse Gas Emissions

Stephen M. Ogle, Cody Alsaker, Jeff Baldock, Martial Bernoux, F. Jay Breidt, Brian McConkey, Kristiina Regina, and Gabriel G. Vazquez-Amabile

This supplement provides additional details about the analysis and sources of data. Data were compiled from 178 studies with 1205 observations of changes in SOC between full tillage and no-till management practices (Supplementary Table S1). A subset of these studies provided additional information to estimate the change in soil mass between full tillage and no-till management, as discussed below. Those studies are notated in Supplementary Table S1.

Supplementary Table S1. List of studies used to fit the semi-parametric mixed effect models. The ΔSOC estimates are by depth in the same order as listed in the depth increments column. All studies were used in the analysis of ΔSOC, but only the studies notated with an 'x' in the soil mass model column provided the data to analyze the difference in soil mass.

Study	Location	Years	Depth Increments (cm)	ΔSOC (tonnes C ha^{-1} yr^{-1})	Soil Mass Model
Al-Kaisi *et al.* 2005	Kanawha, Iowa	6	0-15, 15-30	7.9, 5.7	
	Sutherland, Iowa	6	0-15, 15-30	1.8, -1.8	
	Nashua, Iowa	6	0-15, 15-30	6.9, -4.2	
	Armstrong, Iowa	6	0-15, 15-30	2.0, -0.4	
	Crawfordsville, Iowa	6	0-15, 15-30	7.7, -0.1	
Alvarez *et al.* 1998	Pampa, Arg	15	0-5, 5-10, 10-15, 15-20	2.4, 2.2, 0.2, -0.2	
Alvaro-Fuentes *et al.* 2009	Selvanera, Ebro River Valley, Northeast Spain	17	0-5, 5-10, 10-20	3.5, -1.0, -11.5	x
	Agramunt	13	0-5, 5-10, 10-20	2.7, 1.5, 0.2	
	Penaflor	14	0-5, 5-10, 10-20, 0-5, 5-10, 10-20	3.0, 1.4, 0.2, 1.8, 0.6, 0.5	
Alvaro-Fuentes *et al.* 2014	Agramunt, Lleida, Northeast Spain	11	0-5, 5-10, 10-20, 0-5, 5-10, 10-20, 0-5, 5-	4.2, -1.1, -5.4, -4.0, -3.2, 7.2, 3.6, -1.3,	

			10, 10-20, 20-30, 30-40, 0-5, 5-10, 10-20, 20-30, 0-5, 5-10, 10-20, 20-30	-5.1, -4.1, 3.8, 1.6, 0.2, -1.0, -1.6, 6.2, 1.8, -1.1, -2.2, 6.0, 1.3, 1.3, -2.8	
Angers *et al.* 1995, 1997	Harrington, P.E.I.	8	0-60	-7.9	
	Harrow, Ontario	11	0-60	-0.9	
	La Pocatiere	6	0-60	-20.3	
	Ottawa, Ontario	5	0-60	10.4	
Anken *et al.* 2004	Tanikon, Switzerland	7	0-10, 10-20, 0-10, 10-20, 0-10, 10-20	3.0, 0.0, 5.0, 1.0, 2.0, -3.0	
Balesdent *et al.* 1990	Boigneville	17	0-5, 5-10,10-15, 15-20, 20-25, 25-30	3.4, 1.0, 0.8, 0.5, -0.6, -1.8	x
Bayer *et al.* 2006	Luziania, Goias, Brazil	8	0-2.5, 2.5-5, 5-10, 10-15, 15-20	3.7, 1.3, -1.7, -0.4, -0.3	
	Costa Rica, Mato Grosso, Brazil	5	0-2.5, 2.5-5, 5-10, 10-15, 15-20	2.7, 2.2, -0.5, -0.7, -0.7	
Bayer *et al.* 2002	Eldorado do Sul	9	0-2.5, 2,5-5, 5-7.5, 7.5-12.5, 12.5-17.5	4.5, 2.2, 0.6, 0.3, -0.6	
Beare *et al.* 1994	Athens Georgia	13	0-5, 5-15	5.4, 7.6	x
Bhattacharyya *et al.* 2012	Hawalbagh, Almora, Uttaranchal	9	0-5, 5-15, 15-30	1.3, 1.4, 0.7	
Black *et al.* 1997	Mandan, ND	6	0-7.6, 7.6-15.2, 15.2-30.4, 30.4-60.9, 60.9-91.2, 0-7.6, 7.6-15.2, 15.2-30.4, 0-7.6, 7.6-15.2, 15.2-30.4, 30.4-60.9, 60.9-91.2, 0-7.6, 7.6-15.2, 15.2-30.4, 30.4-60.9, 60.9-91.2, 0-7.6, 7.6-15.2, 15.2-30.4, 30.4-60.9, 60.9-91.2, 0-7.6,	0.7, -0.5, -1.4, -0.6, -2.6, 0.4, -1.1, -1.4, -3.6, -1.2, -3.6, -5.5, -0.1, -11.2, 1.55, -0.7, 2.3, -2.2, -7.3, 3.0, 1.3, 3.6, 5.2, 0.9, 1.9, 0.7, 6.0, 6.1, 3.0, -6.5, -3.9	

			7.6-15.2, 15.2-30.4, 30.4-60.9, 60.9-91.2, 30.4-60.9, 60.9-91.2		
Blanco-Canqui *et al.* 2004	Kingdom City, MO	13	0-10, 10-20, 0-10, 10-20	6.1, 2.5, 1.7, -3.0,	
Blanco-Canqui *et al.* 2011	Hutchinson, KS	23	0-2.5, 2.5-5, 5-10, 10-15, 15-20, 20-30, 30-40, 40-60, 60-80, 80-100	2.4, 0.5, -1.3, -2.0, -1.1, -0.3, -2.8, -3.7, 3.3, 1.2	
	Hays, KS	45	0-2.5, 2.5-5, 5-10, 10-15, 15-20, 20-30, 30-40, 40-60, 60-80, 80-100	1.3, 1.0, 0.5, -0.1, 0.3, -0.2, -0.3, 0.6, -0.8, -0.1	
	Tribune, KS	21	0-2.5, 2.5-5, 5-10, 10-15, 15-20, 20-30, 30-40, 40-60, 60-80, 80-100	1.0, 0.6, -0.2, 0.5, 0.8, 2.3, 1.6, -1.4, 2.0, 1.2	
Boddey *et al.* 2010	Passo Fundo	15	0-30, 30-100	3.8, 12.9	
	Cruz Alta	17	0-30, 30-100, 0-30, 30-100	-4.1, 0.3, 3.6, 5.2	
	Santo Angelo	26	0-30, 30-100, 0-30, 30-100	9.1, 13.0, 1.0, 11.6	
Buschiazzo *et al.* 1998	Buenos Aires, Argentina	7	0-5	5.3	
Buyanovsky *et al.* 1998	Columbia, MO	25	0-20	3.6	
Calegari *et al.* 2008	Pato Branco, Brazil	19	0-5, 5-10, 10-20, 20-30, 30-40, 40-60, 0-5, 5-10, 10-20, 20-30, 30-40, 40-60, 0-5, 5-10, 10-20, 20-30, 30-40, 40-60, 0-5, 5-10, 10-20, 20-30, 30-40, 40-60, 0-5, 5-10, 10-20, 20-30, 30-40, 40-60, 0-5, 5-10, 10-20,	4.1, 0.5, -1.8, -0.3, -0.7, 0.5, 7.2, 2.6, -2.0, -2.0, 1.5, -.4, 5.1, 1.0, -2.3, -3.3, 0.0, 1.4, 7.1, 1.1, -3.7, -1.8, 0.4, 1.7, 8.2, -1.2, -4.7, -3.9, -1.7, -3.1, 4.5, -0.2, -3.6, 3.9, 3.9, 6.2	

			20-30, 30-40, 40-60		
Campbell *et al.* 1996, 1999	Swift Current, Saskatchewan	5	0-7.5, 7.5-15, 0-7.5, 7.5-15, 0-7.5, 7.5-15	1.0, 0.2, 1.3, 0.9, 1.6, -0.3	
	Stewart Valley	8	0-7.5, 7.5-15, 0-7.5, 7.5-15	1.8, 0.3, 3.2, -0.2	x
Carter *et al.* 1988, 1991, 1994, 2002	Charlottetown, PEI	5	0-16	7.8	
Chan *et al.* 1988, 2002, 2003	Wagga Wagga, New South Wales	10	0-5, 5-10, 10-15, 15-20	4.3, 2.9, -1.3, 0.2	
Chaney *et al.* 1985	Headly Hall, Tadcaster, North Yorkshire	5	0-5, 5-10, 10-15, 15-20, 0-5, 5-10, 10-15, 15-20, 0-5, 5-10, 10-15, 15-20	1.5, 0.3, 0.5, 0.5, 2.3, 0.3, 0.8, 0.8, 3.1, 1.7, 0.7, 0.6	
Chen *et al.* 2009	Chenghuang, Linfen, China	11	0-15, 15-30	2.7, -2.1	
Chen *et al.* 2014	Ningxiang, Hunan, China	6	0-5, 5-10, 10-20, 20-30	0.3, -0.0, 2.2, 0.1	x
Clapp *et al.* 2000	Rosemount, MN	6	0-15, 15-30, 0-15, 15-30, 0-15, 15-30, 0-15, 15-30, 0-15, 15-30, 0-15, 15-30, 0-15, 15-30, 0-15, 15-30, 0-15, 15-30, 0-15, 15-30, 0-15, 15-30, 0-15, 15-30, 0-15, 15-30, 0-15, 15-30, 0-15, 15-30, 0-15, 15-30	-0.7, -6.8, -0.5, -9.5, -3.5, -9.8, 0.6, -9.7, 7.3, -6.4, 6.8, -6.5, 6.4, -2.4, 6.9, -8.6, 1.8, -5.7, -1.8, -10.8, 0.3, -10.9, 11.8, -1.1, 1.2, -7.2, 6.7, 13.4, 3.6, -13.2, 3.1, -10.4	
Corazza *et al.* 1999	cerrado region, Federal District, Brazil	15	0-20, 20-40, 40-100	10.0, 4.5, 11.7	
Costantini *et al.* 1996	Marcos Juarez, Cordoba, Arg	6	0-5	2.9	
Dalal *et al.* 1991	Warwick, Queensland	20	0-2.5, 25.-5, 5-10	0.3, 0.4, 0.4	
Denef *et al.* 2007	Passo Fundo, Rio Grande do Sul	15	0-5, 0-20	3.1, 3.8	

Dick *et al.* 1998	Wooster, OH	31	0-30, 0-30, 0-30	1.3, 2.0, 2.0, 5.3, 1.9, -0.5, -0.7, -0.8, 0.0, 0.2, 0.1, 0.1	
Devine *et al.* 2014	Oconee River, Georgia, USA	29	0-5, 5-15, 15-30, 30-50, 50-100	4.8, -0.9, 0.6, 0.8, 0.6	x
Dikgwatlhe *et al.* 2014	Luancheng Agro-Ecosystem E.S., Taihang Mtns, Hebei Province	11	0-5, 5-10, 10-20, 20-30, 30-50	2.7, 2.0, -0.6, -3.3, 1.3	
Dimassi *et al.* 2014	Boigneville, Northern France	41	0-5.2, 5.2-10.8, 10.8-16.4, 16.4-27.9, 27.9-31.4, 31.4-56.6	5.1, 5.7, 4.5, 0.9, 0.7, 0.3	
Dolan *et al.* 2006	Rosemount, Minnesota	22	0-5, 5-10, 10-15, 15-20, 20-25, 25-30, 30-45, 0-5, 5-10, 10-15, 15-20, 20-25, 25-30, 30-45	5.0, 3.8, 0.4, -9.8, -19.1, -3.7, -1.6, 5.9, 4.4, 0.7, -1.0, -7.5, -3.4, -1.6	
Doran *et al.* 1998	Sidney, NE	11	0-7.6, 0-7.6, 7.6-15.2, 7.6-15.2, 0-20, 0-30.5	3.3, 5.2, -0.9, -1.2, -2.3, -1.1, 3.0, 2.7	
Dou *et al.* 2008	Brazos River Floodplain, Texas	20	0-5, 5-15, 15-30, 0-5, 5-15, 15-30	2.2, -1.3, -0.9, 3.7, 1.9, 6.2	
Du *et al.* 2010	Taihang Mountains, China	6	0-5, 5-10, 10-20, 20-30	2.6, 1.4, -1.1, -0.9	
Du *et al.* 2015	Taihang Mountains, China	6	0-5, 5-10, 10-20, 20-30, 30-40, 40-50	2.1, 0.9, -1.4, -0.8, -0.6, 0.1	
Duiker & Lal 1999	Columbus, OH	7	0-10	4.0	
Edwards *et al.* 1992	Crossville, AL	11	0-5, 5-10, 10-20, 0-5, 5-10, 10-20, 0-5, 5-10, 10-20	3.7, 3.9, 0.1, 6.8, 4.5, 1.3, 4.9, 4.9, -0.2	
Eghball *et al.* 1994	Lincoln, NE	11	0-30	5.2	
Fabrizzi *et al.* 2003, 2009	Balcarce Experiment Station, Argentina	7	0-7.5, 7.5-15	4.5, 0.5	
	Cruz Alta, Brazil	20	0-5, 5-15, 15-30	6.1, -0.8, -0.7	
	Entre Rios, Argentina	9	0-5, 5-15, 15-30	3.0, -3.8, 0.2	

	Manhattan, Kansas	15	0-5, 5-15, 15-30	3.6, -1.3, -2.4	
Fan *et al.* 2014	Woodslee, Ontario, Canada	30	0-10, 10-20, 20-30, 30-40, 40-60	3.2, -2.6, -1.9, -1.7, -2.4	x
Feiziene *et al.* 2011	Lithuania	11	0-10, 0-10	4.3, 4.9	
Fleige *et al.* 1974	Gottingen I	5	0-5, 5-10, 10-15, 15-20, 20-30	4.1, 0.9, 0.7, 0.3, 0.2	
	Gottingen II	5	0-5, 5-10, 10-15, 15-20, 20-30	3.1, 2.1, 2.0, 1.8, 0.2	
	Gottingen III	6	0-5, 5-10, 10-15, 15-20, 20-30	2.8, 0.7, 0.3, 0.3, 0.0	
Franzluebbers *et al.* 1996	Alberta	6	0-20	2.7	
	British Columbia	7	0-20, 0-20	-0.7, -3.3	
Franzluebbers *et al.* 2013	Watkinsville, Georgia	5	0-20, 20-40, 40-60, 60-90, 90-120, 120-150, 0-20, 20-40, 40-60, 60-90, 90-120, 120-150	4.3, -0.5, 2.5, 0.1, 0.4, 0.3, 6.8, -2.9, -1.2, 0.2, -0.6, 1.2	
Franzluebbers *et al.* 1998	south-central Texas	10	0-5, 5-12.5, 12.5-20, 0-5, 5-12.5, 12.5-20, 0-5, 5-12.5, 12.5-20, 0-5, 5-12.5, 12.5-20	2.2, -0.9, -0.7, 2.8, -0.4, -0.0, 3.5, -0.9, -0.9, 3.4, -1.1, -1.0	
Freitas *et al.* 2000	regiao centro-sul de Goias, nas esacoes experimentais de emater, antiga Emgopa	5	0-10, 10-20, 20-40	1.5, -0.4, -2.9	
Gal *et al.* 2007	West Lafayette, IN	28	0-5, 5-15, 15-30, 30-50, 50-75, 75-100	9.0, 8.5, 5.5, -10.7, -1.7, -1.1	x
Garcia-Prechac *et al.* 2004	Uruguay	7	0-12	6.0	
Green *et al.* 2007	Sete Lagoas, Brazil	5	0-5, 5-20, 20-30	4.6, -0.7, -0.5	
Gwenzi *et al.* 2009	Save Valley Experiment Station, Chipinge town	5	0-15, 15-30, 30-45, 45-60	6.2, 12.1, 12.8, 13.8	

Halvorson *et al.* 1997	Akron, CO	15	0-2.5, 2.5-5, 5-10, 10-20	0.5, 0.6, 0.4, 0.3	x
Havlin *et al.* 1997	Manhattan, KS	11	0-2.5, 2.5-7.5, 7.5-15, 0-2.5, 2.5-7.5, 7.5-15, 0-2.5, 2.5-7.5, 7.5-15	1.1, 0.2, 0.3, 2.3, 0.7, -0.1, 2.1, -0.5, -0.7	
Hernanz *et al.* 2002	Alcala de Henares, Madrid	11	0-10, 10-20, 20-30, 30-40	4.6, -0.2, -1.0, -1.6	
	Alcala de Henares, Madrid, Spain	6	0-10, 10-20, 20-30, 30-40, 0-10, 10-20, 20-30, 30-40	6.3, -0.1, -0.8, -3.1, 4.1, -0.4, -0.7, 0.0	
Higashi *et al.* 2014	Kanto region, Ibaraki	9	0-2.5, 2.5-7.5, 7.5-15, 15-30, 0-2.5, 2.5-7.5, 7.5-15, 15-30, 0-2.5, 2.5-7.5, 7.5-15, 15-30	1.5, 2.3, 0.9, 0.1, 0.7, 4.4, 2.3, -0.1, 4.0, 4.5, -0.2, -3.2	
Hou *et al.* 2011	Yucheng Comprehensive Experiment Station of China Academy of Science	5	0-5, 2-60	1.9, -6.3	
Hussain *et al.* 1999	Dixon Springs	6	0-15, 0-15	6.5, 3.9	
Ismail *et al.* 1994	Lexington, KY	20	0-5, 5-15, 15-30, 0-5, 5-15, 15-30, 0-5, 5-15, 15-30, 0-5, 5-15, 15-30	6.4, 1.4, -1.2, 6.2, 2.8, -6.9, 7.2, 0.7, -5.4, 8.1, 0.6, -3.8	x
Jagadamma *et al.* 2010	Wooster, Ohio	41	0-7.5, 7.5-15, 15-30, 30-45	8.6, 3.1, -4.9, -0.5	
Jarecki *et al.* 2005	South Charleston, Ohio	41	0-5, 5-15, 15-30, 30-50, 50-80	9.3, -2.0, -1.2, -2.7, 2.7	x
	Hoytville, Ohio	16	0-5, 5-15, 15-30, 30-50, 50-80	-0.1, -1.6, -0.4, -3.7, 2.0	
Jemai *et al.* 2012, 2013	Mateur, Tunisia	7	0-10, 10-20, 20-30, 30-40, 40-50	4.4, 0.9, -4.4, -6.2, -5.5	x
Karlen *et al.* 1994	Nashua, IA	15	0-20	18.4	
	Lancaster, WI	10	0-2.5, 2.5-5, 5-15, 15-22.5, 22.5-30, 30-45, 45-60	6.2, 1.5, 8.8, 6.4, 2.3, 0.0, 0.0	

Kumar *et al.* 2012, 2014	Columbus, Ohio	18	0-10, 10-20, 20-40, 40-60	5.3, 6.2, 1.2, 3.5	
Lal 1998	Ibadan	7	0-5, 5-10	2.8, 0.6	
Lammerding *et al.* 2010	Alcala de Henares, Spain	12	0-7.5, 7.5-15, 0-7.5, 7.5-15	3.0, 0.7, 3.2, -0.7	x
Larney *et al.* 1997	Lethbridge, Alberta	7	0-15, 0-15, 0-15, 0-15, 0-15, 0-15	2.0, 0.0, 0.8, 2.0, 0.0, 0.8	
Laudicina *et al.* 2014	Sicily	18	0-15	0.8	
Liang *et al.* 2011	Dehui, China	5	0-5, 5-10, 10-20-, 20-30	-0.3, -1.0, -0.4, -2.1	x
Liu *et al.* 2014	Shanxi Province, Linfen County	17	0-5, 5-10, 10-20-, 20-30, 40-50, 50-60	5.0, 2.3, 0.4, -0.7, -2.1, -0.6, -0.4	
Lopez-Bellido *et al.* 2009, 2017	Cordoba, Spain	6, 27	0-90, 0-90, 0-90, 0-90, 0-90, 0-90	0.8, 10.9, 8.3, 8.8, 6.0, 8.1	x
Lopez-Fando *et al.* 2007, 2009	CSIC farm "La Higueruela", Toledo, Spain	11	0-5, 5-10, 10-20, 20-30, 0-5, 5-10, 10-20, 20-30	4.4, 1.4, 2.7, 3.8, 5.6, 0.1, -2.1, 0.4	
	Madrid, Spain	10	0-5, 5-10, 10-20, 20-30, 0-5, 5-10, 10-20, 20-30	5.8, 3.1, 0.7, -1.1, 7.6, 4.2, -0.2, -1.3	x
	Santa Olalla, Toledo	5	0-5, 5-10, 10-20, 20-30, 0-5, 5-10, 10-20, 20-30, 0-5, 5-10, 10-20, 20-30, 0-5, 5-10, 10-20, 20-30, 0-5, 5-10, 10-20, 20-30, 0-5, 5-10, 10-20, 20-30, 0-5, 5-10, 10-20, 20-30, 0-5, 5-10, 10-20, 20-30, 0-5, 5-10, 10-20, 20-30, 0-5, 5-10, 10-20, 20-30	5.0, 1.8, -2.4, 0.1, 4.5, 0.1, 4.6, -1.4, 4.8, 0.9, 2.7, 0.8, 5.3, 0.7, 8.6, -0.6, 6.0, 3.1, -2.7, -2.7, 4.9, 4.9, 0.3, -2.0, 5.6, 3.3, -1.2, -4.4, 6.5, 3.6, -2.8, -0.4, 7.2, 4.7, -2.7, -2.5, 7.7, 4.0, -0.8, -4.0, 8.6, 3.7, -1.4, -1.9, 7.6, 3.5, -1.8, -3.2	

Lou *et al.* 2012	Jianping county	12	0-5, 5-10, 10-20, 20-40, 40-60, 60-80, 80-100	1.4, -0.7, -3.4, -7.7, -7.2, -3.2, -3.0	
	Changtu county	5	0-5, 5-10, 10-20, 20-40, 40-60, 60-80, 80-100	1.8, 0.3-2.3, 0.3, 0.7, -0.1, 0.4	
Martinez *et al.* 2013	University of Chile, Santiago, Chile	9	0-2, 2-5, 5-15	2.2, 1.2, 0.6	
Martin-Lammerding *et al.* 2013	INIA farm, NE of Madrid	12	0-15, 0-15	3.7, 2.5	
Mielke *et al.* 1986	Elwood, IL	6	0-7.5, 7.5-15, 15-30	7.5, 1.6, -3.1	
	Waseca, MN	6	0-7.5, 7.5-15, 15-30, 0-7.5, 7.5-15	5.1, -4.3, -0.9, 9.1, -2.6	
Mrabet *et al.* 2001	Sidi El Aydi, Morrocco	11	0-2.5, 2.5-7, 7-20	2.7, 0.3, 0.3	
Murage *et al.* 2006	Delhi, Ontario	10	0-5, 0-10, 0-15, 0-20	2.0, 1.3, 0.1, -0.6	
Nyborg *et al.* 1995	Breton, Alberta	11	0-5, 5-10, 10-15, 0-5, 5-10, 10-15, 0-5, 5-10, 10-15	2.6, 1.0, -0.3, 3.8, 0.7, 3.0, 1.4, -0.3, -0.8	
	Ellerslie, Alberta	11	0-5, 5-10, 10-15, 0-5, 5-10, 10-15, 0-5, 5-10, 10-15	2.1, 1.0, -1.2, -0.5, 0.0, -1.3, 1.4, 0.9, -1.5	
Olson *et al.* 2005	Southern Illinois	6	0-5, 5-15, 15-30, 0-5, 5-15, 15-30, 0-5, 5-15, 15-30, 30-45, 45-60, 60-75	3.9, 1.1, 0.0, 5.5, 1.1, -1.7, 4.5, 2.4, 0.5, 0.1, 0.7, 1.1	x
Page *et al.* 2013	Mackay	10	0-30	1.4	
	Ingham	10	0-30	-2.2	
	Bundaberg	5	0-30	2.5	
	Biloela	26	0-10, 0-30	-2.1, -11.8	
	Hermitage	40	0-10, 10-30	0.5, -1.2	
Paustian *et al.*, unpublished	Griffin, GA	18	0-20, 20-25, 25-50	12.1, -3.9, -2.5	
	Hickory Corners, MI	6	0-20, 20-25, 25-50	6.7, 0.7, 5.7	
	Hoytville, OH	29	0-20, 20-25, 25-50	20.2, -4.3, -9.9	

	South Charleston, OH	30	0-20, 20-25, 25-50	11.5, 2.0	
	Sterling, CO	7	0-20, 20-25, 25-50	1.3, 0.1, -0.5	
Pierce *et al.* 1997	East Lansing	7	0-5, 5-10, 0-5, 5-10	4.4, 1.8, 3.6, 0.0	
Plaza-Bonilla *et al.* 2011	Agramunt	17	0-5, 5-10, 10-20, 20-30, 30-40	3.8, 1.6, 0.1, -1.8, -3.1	x
	Selvanera	20	0-5, 5-10, 10-20, 20-30, 30-40	2.1, -0.7, -2.2, -0.1, 0.8	x
Robertson *et al.* 2015	Walpeup	28	30	0.5, 1.4, 1.7, 1.3	
Rhoton *et al.* 1993	Auburn, AL	5	7.6-15.2, 0-2.5, 2.5-7.6	1.3, 1.0, 0.9	
	Jackson, TN	9	0-2.5, 2.5-7.6, 7.6-15	1.8, -1.3, -1.8	
	Verona, MS	5	0-2.5, 2.5-7.6, 7.6-15	2.2, 0.1, -1.0	
	Watkinsville, GA	15	0-2.5, 2.5-7.6, 7.6-15	10.0, 0.4, -2.8	
Sa *et al.* 2001	Ponta Grossa	22	0-2.5, 2.5-5, 5-10, 10-20, 20-40	9.5, 5.7, 2.6, 0.5, 0.9	
Sa *et al.* 2014	Ponta Grossa, Parana	13	0-2.5, 2.5-5, 5-10, 10-20, 20-40, 0-2.5, 2.5-5, 5-10, 10-20, 20-40	4.4, 2.9, 1.4, -1.3, 13.0, 5.6, 4.2, 3.3, 0.2, 12.4	x
Saffigna *et al.* 1989	Biloela, Queensland, Australia	5	0-10	-2.8	
Sainju *et al.* 2007	Culbertson, Montana	21	0-5, 5-20	0.6, 1.1	x
Sainju *et al.* 2006	Havre, MT	5	0-5, 5-20, 0-5, 5-20, 0-5, 5-20, 0-5, 5-20, 0-5, 5-20	1.0, 0.5, 0.7, -1.2, 1.2, -0.5, 1.9, 1.1, 1.0, 0.1	
	Fort Valley, GA	7	0-10, 10-30, 30-60, 60-90, 90-120, 0-10, 10-30, 30-60, 60-90, 90-120, 0-10, 10-30, 30-60, 60-90, 90-120, 0-10,	2.6, 1.6, 0.9, 1.3, 1.5, 2.2, 1.2, 1.5, 0.3, -0.7, 2.7, 0.5, 0.8, 2.9, 0.0, 1.1, 1.2, 1.1, 0.9, 0.3	

			10-30, 30-60, 60-90, 90-120		
Sainju *et al.* 2014	Nesson Valley, North Dakota	6	0-5, 5-10, 10-20, 20-50, 50-85, 0-5, 5-10, 10-20, 20-50, 50-85	1.0, -0.5, -0.9, -0.2, 2.3, 0.2, -0.6, -0.5, 1.5, -4.0	
Sainju *et al.* 2008	Belle Mina, AL	9	0-10, 10-20, 0-10, 10-20	1.2, -0.5, -1.0, 2.1	
Salinas-Garcia *et al.* 1997	Texas A&M University Agricultural Research and Exension Center, Texas	16	0-5, 5-12.5, 12.5-20	3.2, 1.2, -1.0	
Salinas-Garcia *et al.* 2002	Apatzingan, Mexico	6	0-5, 5-10, 10-15	1.6, 0.0, 0.0	
	Casas Blancas, Mexico	6	0-5, 5-10, 10-15	12.0, 5.0, 0.5	
	Morelia, Mexico	6	0-5, 5-10, 10-15	2.5, 0.0, 0.0	
	Tepatitlan, Mexico	6	0-5, 5-10, 10-15	4.0, 0.0, 0.0	
Salvo *et al.* 2010	Paysandu, Uruguay	10	0-3, 3-6, 6-12, 12-18, 0-3, 3-6, 6-12, 12-18	2.8, 0.1, -0.5, -1.7, 3.1, 0.1, 0.3, -1.0	
Schomberg *et al.* 1998	Bushland, TX	12	0-2, 2-4, 4-8, 0-2, 2-4, 4-8	0.4, 0.4, 0.2, 0.7, 0.6, 0.2	
Sheehy *et al.* 2013	Jokioinen, Finland	9	0-20, 0-20	3.7, 0.3	x
	Vihti, Finland	9	0-20, 0-20	-10.7, -2.5	x
	Sakyla, Finland	11	0-20	-2.5	x
Shi *et al.* 2011	Woodslee, Ontario	16	0-10, 0-20, 0-30	3.6, -1.2, -4.7	
Shrestha *et al.* 2015	Aas, Ostland, Norway	26	0-10, 0-30	12.1, 1.3	
Sombrero *et al.* 2010	Torrepadierne farm	10	0-10, 0-20, 0-30, 0-10, 0-20, 0-30, 0-10, 0-20, 0-30, 0-10, 0-20, 0-30, 0-10, 0-20, 0-30	7.9, 11.2, 15.3, 7.6, 10.4, 13.6, 8.9, 11.4, 15.0, 7.0, 9.1, 12.3, 5.0, 6.1, 9.5	
Sun *et al.* 2011	Dundee, Scotland	5	0-5, 0-10, 0-20, 0-30, 0-40, 0-60	4.4, 62., 5.5, 4.5, -3.2, 0.8	x
Thomas *et al.* 2007	Goondawindi	9	0-10, 10-20, 20-30	1.9, -0.3, 0.1	
Tian *et al.* 2013	Tai'an, China	10	0-30, 0-30	3.2, 6.3	

Tivet *et al.* 2013	Lucas do Rio Verde	8	0-20, 20-40, 40-100, 0-20, 20-40, 40-100, 0-20, 20-40, 40-100, 0-20, 20-40, 40-100, 0-20, 20-40, 40-100, 0-20, 20-40, 40-100	10.4, 3.3, 1.4, 5.7, 4.0, 0.3, 3.9, 1.9, 0.3, 3.9, 5.7, 2.5, 9.5, 6.3, 0.6, 6.9, 4.7, 2.9	
Ussiri *et al.* 2009	OARDC, Charleston, Ohio	43	0-15, 15-30	39.5, -4.3	
VandenBygaart *et al.* 2002	Harrington	20	0-15, 15-30, 30-45	3.0, -3.0, 4.0	
	Acadie	13	0-15, 15-30, 30-45, 45-60	4.8, -4.8, 1.3, 0.4	
	elora	25	0-15, 15-30, 30-45, 45-60, 0-15, 15-30, 30-45, 45-60	3.0, -4.0, -3.0, -1.0, 0.0, -3.0, -2.0, -1.0	
	Woodslee	12	0-15, 15-30, 30-45, 45-60	-1.0, 1.0, 4.0, 0.0	
	Woodslee	22	0-15, 15-30, 30-45, 45-60	3.0, -5.0, -4.0, -1.0	
	Scott	24	0-15, 15-30, 30-45, 0-15, 15-30, 30-45	6.4, 32, 0.8, 5.4, 2.3, -0.8	
	Ellerslie	26	0-15, 15-30, 30-45	0.1, 18.0, 4.0	
	Breton	25	0-15, 15-30, 30-45	2.0, 1.0, 0.0	
Varvel *et al.* 2011	Rogers Memorial Farm, Lincoln, Nebraska	24	0-7.5, 7.5-15, 15-30, 0-7.5, 7.5-15, 15-30, 0-7.5, 7.5-15, 15-30, 0-7.5, 7.5-15, 15-30, 30-60, 60-90, 90-120, 120-150	7.6, 1.4, 3.0, 5.2, 1.2, 4.7, 4.0, 1.6, 1.9, 5.9, 3.0, 12.9, 10.1, 6.2, 1.6	x
Viaud *et al.* 2010	Kerguehennec Experimental Station	8	0-5, 5-15, 15-20, 20-25, 25-30, 30-40	1.6, 1.3, -1.1, -0.4, 0.1, 0.5	
Wander *et al.* 1998	DeKalb, IL	10	0-5, 5-15, 15-30	3.8, -0.4, 0.6	x
	Monmouth, IL	10	0-5, 5-15, 15-30	4.0, -0.5, 0.7	x

	Perry, IL	10	0-5, 5-15, 15-30	1.1, -0.7, 2.1	x
Wang *et al.* 2006	Hermitage Research Station, Queensland, Australia	33	0-10, 10-20	0.3, -0.4	
Wright *et al.* 2004	Brazos River flood plain, Texas	20	0-5, 5-15, 0-5, 5-15, 0-5, 5-15	4.3, -0.1, 3.4, -1.9, 3.7, 4.4	
Xu *et al.* 2013	Ningxiang, Hunan Province	4.5	0-5, 5-10, 10-20-, 20-30, 30-40, 40-60, 60-80	0.8, 0.7, -1.4, -5.4, 1.0, 0.1, -0.5	x
Yang *et al.* 2001	Southern Ontario Canada	19	0-5, 5-10, 10-20, 20-30, 30-40, 40-50, 50-60, 0-5, 5-10, 10-20, 20-30, 30-40, 40-50, 50-60, 0-5, 5-10, 10-20, 20-30, 30-40, 40-50, 50-60	3.2, 4.9, 12.4, -1.1, -6.6, -13.4, 3.1, 6.0, 2.3, -0.4, -3.6, -2.6, -0.1, 2.6, 12.1, 11.8, 17.7, 26.1, 6.7, 5.1, 1.1	x
Yang *et al.* 1999	Urbana, IL	11	0-5, 5-10, 10-20, 20-30, 30-40, 40-50, 50-70, 70-90	2.6, 0.4, 1.5, -3.6, 0.3, 0.3, -1.1, -0.9	x
Zhang *et al.* 2007	Delta Junction, Alaska, USA	20	0-5, 5-10	1.0, -0.5	
Zhang *et al.* 2017	Fengqiu National Experimental Station	9	0-5, 5-10, 10-20, 0-5, 5-10, 10-20	1.2, 0.3, -1.6, 0.7, -1.0, -1.6	

The difference in soil mass was predicted using a semi-parametric modeling approach for loamy, silty and clayey soils (i.e., differences in mass for sandy soils were not significant). The soil mass difference model was applied by predicting the change in mass to a specified depth (the depth is based on significant differences in SOC between the management practices, See Table 1). The change in mass ($\Delta Mass$) is estimated using the following equation:

$$\Delta Mass = Mass_{NT} - Mass_{FT}, \quad \text{(S1)}$$

where soil mass under full tillage ($Mass_{FT}$) is subtracted from the soil mass under no-till management ($Mass_{NT}$). The adjusted estimate (ΔSOC_{adj}) for mass equivalency was estimated using the following equation,

$$\Delta SOC_{adj} = \Delta SOC - (\Delta Mass * \%OC_i), \qquad \text{(S2)}$$

where the estimated difference in soil mass ($\Delta Mass$) is multiplied by the percent SOC ($\%OC_i$) for the tillage practice with greater mass at the specified depth, and subtracted from the unadjusted estimate (ΔSOC_{adj}). Supplementary Figure S1 is a plot of the modeled change in the mass for loamy, silty and clayey soils between no-till and full tillage management across depths from 0 to 100 cm.

Supplementary Figure S1. Change in the mass (g cm^{-2}) between no-till and full tillage management across depths from 0 to 100 cm for loamy, silty, and clayey soils. The red lines represent the observed changes in mass at the depth range of each line, and the blue shading is a 95% confidence interval for the estimated change in mass (Δmass = mass$_{\text{no-till}}$ – mass$_{\text{full till}}$).

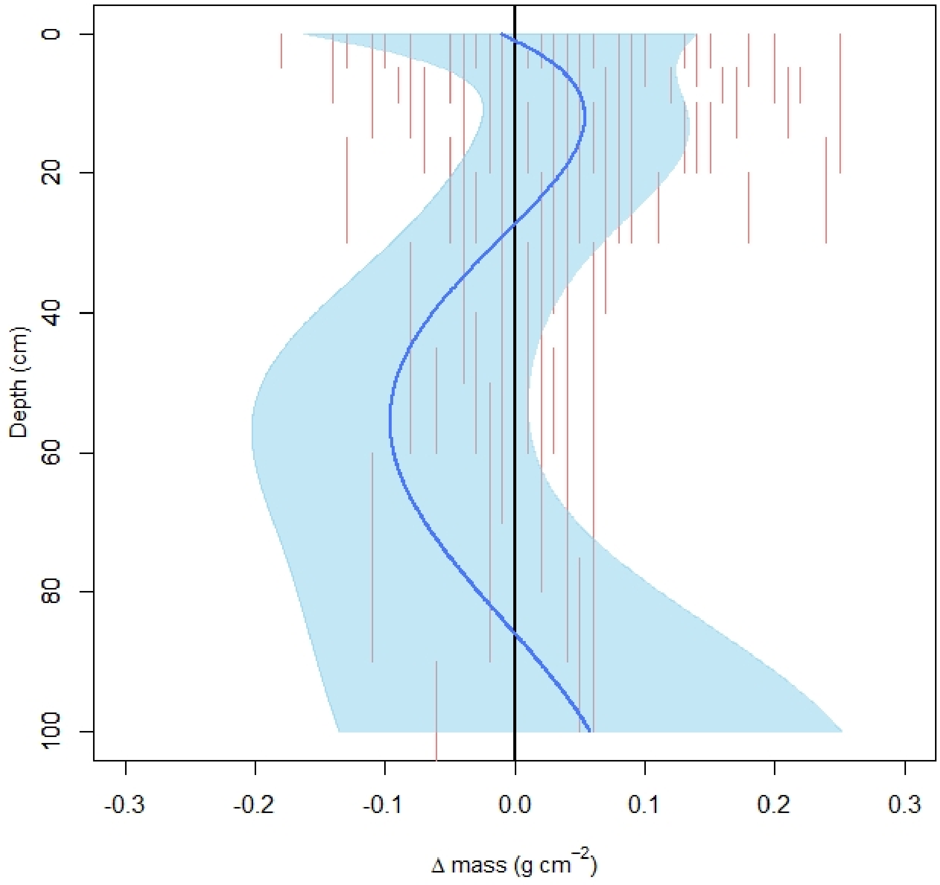

References

1 Al-Kaisi , M., Yin, X. & Licht, M. Soil carbon and nitrogen changes as affected by tillage system and crop biomass in a corn-soybean rotation. *Applied Soil Ecology* **30**, 174-191 (2005).

2 Al-Kaisi, M., Yin, X. & Licht, M. Soil carbon and nitrogen changes as influenced by tillage and cropping systems in some Iowa soils. Agriculture, Ecosystems & Environment *Agriculture Ecosystems & Environment* **105**, 635-647 (2005).

3 Alvarez, C. R., Alvarez, R., Grigera, M. S. & Lavado, R. S. Associations between organic matter fractions and the active soil microbial biomass. *Soil Biology & Biochemistry* **30**, 767-773 (1998).

4 Alvaro-Fuentes, J. *et al.* Soil aggregation and soil organic carbon stabilization: Effects of management in semiarid Mediterranean agroecosystems. *Soil Science Society of America Journal* **73**, 1519-1529 (2009).

5 Alvaro-Fuentes, J., Plaza-Bonilla, D., Arrue, J. L., Lampurlanes, J. & Cantero-Martinez, C. Soil organic carbon storage in a no-tillage chronosequence under Mediterranean conditions. *Plant and Soil* **376**, 31-41 (2014).

6 Angers, D. A. *et al.* Impact of tillage practices on organic carbon and nitrogen storage in cool, humid soils of eastern Canada. *Soil Tillage Research* **41**, 191-201 (1997).

7 Angers, D. A., Voroney, R. P. & Côté, D. Dynamics of soil organic matter and corn residues affected by tillage practices. *Soil Science Society of America Journal* **59**, 1311-1315 (1995).

8 Anken, T. *et al.* Long-term tillage system effects under moist cool conditions in Switzerland. *Soil & Tillage Research* **78**, 171-183 (2004).

9 Balesdent, J., Mariotti, A. & Boisgontier, D. Effect of tillage on soil organic carbon mineralization estimated from 13C abundance in maize fields. *Journal of Soil Science* **41**, 587-596 (1990).

10 Bayer, C., Martin-Neto, L., Mielniczuk, J., Pavinato, A. & Dieckow, J. Carbon sequestration in two Brazilian Cerrado soils under no-till. *Soil & Tillage Research* **86**, 237-245 (2006).

11 Bayer, C., Mielniczuk, J., Martin-Neto, L. & Ernani, P. R. Stocks and humification degree of organic matter fractions as affected by no-tillage on a subtropical soil. *Plant and Soil* **238**, 133-140 (2002).

12 Beare, M. H., Hendrix, P. F. & Coleman, D. C. Water-stable aggregates and organic matter fractions in conventional- and no-tillage soils. *Soil Science Society of America Journal* **58**, 777-786 (1994).

13 Bhattacharyya, R. *et al.* Tillage and irrigation effects on soil aggregation and carbon pools in the Indian Sub-Himalayas. *Agronomy Journal* **105**, 101-112 (2013).

14 Black, A. L. & Tanaka, D. L. *A conservation tillage-cropping systems study in the northern Great Plains of the United States*. 335-342 (CRC Press, 1997).

15 Blanco-Canqui, H., Gantzer, C. J., Anderson, S. H. & Alberts, E. E. Tillage and crop influences on physical properties for an Epiaqualf. *Soil Science Society of America Journal* **68**, 567-576 (2004).

16 Blanco-Canqui, H., Schlegel, A. J. & Heer, W. F. Soil-profile distribution of carbon and associated properties in no-till along a precipitation gradient in the central Great Plains. *Agriculture Ecosystem & Environment* **144**, 107-116 (2011).

17 Boddey, R. *et al.* Carbon accumulation at depth in Ferralsols under zero-till subtropical agriculture. *Global Change Biology* **16**, 748-795 (2010).

18 Buschiazzo, D. E., Panigatti, J. L. & Unger, P. W. Tillage effects on soil properties and crop production in the subhumid and semiarid Argentinean Pampas. *Soil & Tillage Research* **49**, 105-116 (1998).

19 Buyanovsky, G. A. & Wagner, G. H. Carbon cycling in cultivated land and its global significance. *Global Change Biology* **4**, 131-141 (1998).

20 Calegari, A. *et al.* Impact of long-term no-tillage and cropping system management on soil organic carbon in an Oxisol: A model for sustainability. *Agron Journal* **100**, 1013-1019 (2008).

21 Campbell, C. A., Biederbeck, V. O., McConkey, B. G., Curtin, D. & Zentner, R. P. Soil quality-effect of tillage and fallow frequency. Soil organic matter quality as influenced by tillage and fallow frequency in a silt loam in southwestern Saskatchewan. *Soil Biology and Biochemistry* **31**, 1-7 (1999).

22 Campbell, C. A., McConkey, B. G., Zentner, R. P., Selles, F. & Curtin, D. Long-term effects of tillage and crop rotations on soil organic C and total N in a clay soil in southwestern Saskatchewan. *Canadian Journal of Soil* **76**, 395-401 (1996).

23 Carter, M. R. Evaluation of shallow tillage for spring cereals on a fine sandy loam. 2. Soil physical, chemical and biological properties. *Soil & Tillage Research* **21**, 37-52 (1991).

24 Carter, M. R., Johnston, H. W. & Kimpinski, J. Direct drilling and soil loosening for spring cereals on a fine sandy loam in Atlantic Canada. *Soil & Tillage Research* **12**, 365-384 (1988).

25 Carter, M. R., Mele, P. M. & Steed, G. R. The effects of direct drilling and stubble retention on water and bromide movement and earthworm species in a duplex soil. *Soil Science* **157**, 224-231 (1994).

26 Carter, M. R., Sanderson, J. B., Ivany, J. A. & White, R. P. Influence of rotation and tillage on forage maize productivity, weed species, and soil quality of a fine sandy loam in the cool-humid climate of Atlantic Canada. *Soil & Tillage Research* **67**, 85-98 (2002).

27 Chan, K. Y., Heenan, D. P. & Oates, A. Soil carbon fractions and relationships to soil quality under different tillage and stublle management. *Soil & Tillage Research* **63**, 133-139 (2002).

28 Chan, K. Y., Heenan, D. P. & So, H. B. Sequestration of carbon and changes in soil quality under conservation tillage on light-textured soils in Australia: a review. *Australian Journal of Experimental Agriculture* **43**, 325-334 (2003).

29 Chan, K. Y. & Mead, J. A. Surface physical properties of a sandy loam soil under different tillage practices. *Australian Journal of Soil Research* **26**, 549-559 (1988).

30 Chaney, B. K., Hodson, D. R. & Braim, M. A. The effects of direct drilling, shallow cultivation and ploughing on some soil physical properties in a long-term experiment on spring barley. *Journal of Agriculture Science (Camb)* **104**, 125-133 (1985).

31 Chen, H. *et al.* Effects of 11 years of conservation tillage on soil organic matter fractions in wheat monoculture in Loess Plateau of China. *Soil & Tillage Research* **106**, 85-94 (2009).

32 Chen, H., Marhan, S., Billen, N. & Stahr, K. Soil organic-carbon and total nitrogen stocks as affected by different land uses in Baden-Wurttemberg (southwest Germany). *Journal of Plant Nutrition and Soil Science* **172**, 32-42 (2009).

33 Chen, Z. *et al.* Tillage impacts on net carbon flux in paddy soil of the Southern China. Journal of Cleaner Production. *Journal of Cleaner Production* **103**, 70-76 (2015).

34 Clapp, C. E., Allmaras, R. R., Layese, M. F., Linden, D. R. & Dowdy, R. H. Soil organic carbon and 13C abundance as related to tillage, crop residue, and nitrogen fertilization under continuous corn management in Minnesota. *Soil & Tillage Research* **55**, 127-142 (2000).

35 Corazza, E. J., Silva, J. E., Resck, D. V. S. & Gomes, A. C. Behaviour of different management systems as a source or sink of carbon in relation to cerrado vegetation. *Revista Brasileira de Ciencia do Solo* **23**, 425-432 (1999).

36 Costantini, A., Cosentino, D. & Segat, A. Influence of tillage systems on biological properties of a Typic Argiudoll soil under continuous maize in central Argentina. *Soil & Tillage Research* **38**, 265-271 (1996).

37 Dalal, R. C. Long-term effects of no-tillage, crop residue, and nitrogen application on properties of a Vertisol. *Soil Science Society of America Journal* **53**, 1511-1515 (1989).

38 Dalal, R. C., Henderson, P. A. & Glasby, J. M. Organic matter and microbial biomass in a vertisol after 20 yr of zero tillage. *Soil Biology and Biochemistry* **23**, 435-441 (1991).

39 Denef, K., Zotarelli, L.., Boddey, R. & Six, J. Microaggregate-associated carbon as a diagnostic fraction for management-induced changes in soil organic carbon in two Oxisols. *Soil Biology and Biochemistry* **39**, 1165-1172 (2007).

40 Devine, S., Markewitz, D., Hendrix, P. & Coleman, D. Soil aggregates and associated organic matter under conventional tillage, no-tillage, and forest succession after three decades. *PLoS ONE* **9** (2014).

41 Dikgwatlhe, S. B., Chen, Z., Lal, R., Zhang, H. & Chen, R. Changes in soil organic carbon and nitrogen as affected by tillage and residue management under wheat-maize cropping system in the North China Plain. *Soil & Tillage Research* **144**, 110-118 (2014).

42 Dimassi, B. *et al.* Long-term effect of contrasted tillage and crop management on soil carbon dynamics during 41 years. *Agriculture Ecosystem & Environment* **188**, 134-146 (2014).

43 Dolan, M. S., Clapp, C. E., Allmaras, R. R., Baker, J. M. & Molina, J. A. E. Soil organic carbon and nitrogen in a Minnesota soil as related to tillage, residue and nitrogen management. *Soil & Tillage Research* **89**, 221-231 (2006).

44 Doran, J. W., Elliott, E. T. & Paustian, K. Soil microbial activity, nitrogen cycling, and long-term changes in organic carbon pools as related to fallow tillage management. *Soil Tillage Research* **49**, 3-18 (1998).

45 Dou, F., Wright, A. & Hons, F. Sensitivity of labile soil organic carbon to tillage in wheat-based cropping systems. *Soil Science Society of America Journal* **72**, 1445-1453 (2008).

46 Du, Z., Ren, T. & Hu, C. Tillage and residue removal effects on soil carbon and nitrogen storage in the North China Plain. *Soil Science Society of America Journal* **74**, 197-202 (2010).
47 Du, Z., Ren, T., Hu, C. & Zhang, Q. Transition from intensive tillage to no-till enhances carbon sequestration in microaggregates of surface soil in the North China Plain. *Soil & Tillage Research* **146**, 26-31 (2015).
48 Duiker, S. W. & Lal, R. Crop residue and tillage effects on carbon sequestration in a Luvisol in central Ohio. *Soil & Tillage Research* **52**, 73-81 (1999).
49 Edwards, J. H., Woods, C. W., Thurlow, D. L. & Ruf, M. E. Tillage and crop rotation effects on fertility status of a Hapludult soil. *Soil Science Society of America Journal* **56**, 1577-1582 (1992).
50 Eghball, B., Mielke, L. N., McCallister, D. L. & Doran, J. W. Distribution of organic carbon and inorganic nitrogen in a soil under various tillage and crop sequences. *Soil & Water Conservation* **49**, 201-205 (1994).
51 Fabrizzi, K. P., Moron, A. & Garcia, F. O. Soil carbon and nitrogen organic fractions in degraded vs. non-degraded Mollisols in Argentina. *Soil Science Society of America Journal* **67**, 1831-1841 (2003).
52 Fabrizzi, K. P. *et al.* Protection of soil organic C and N in temperate and tropical soils: effect of native and agroecosystems. *Biogeochemistry* **92**, 129-143 (2009).
53 Fan, R. Q., Yang, X. M., Drury, C. F., Reynolds, W. D. & Zhang, X. P. Spatial distributions of soil chemical and physical properties prior to planting soybean in soil under ridge-, no- and contentional-tillage in a maize-soybean rotation. *Soil Use and Management* **30**, 414-422 (2014).
54 Feiziene, D. *et al.* Long-term influence of tillage and fertilization on net carbon dioxide exchange rate on two soils with different textures. *Journal of Environmental Quality* **40**, 1787-1796 (2011).
55 Fleige, H. & Baeumer, K. Effect of zero-tillage on organic carbon and total nitrogen content, and their distribution in different N-fractions in loessial soils. *Agro-Ecosystems* **1**, 19-29 (1974).
56 Franzleubbers, A. J., Hons, F. M. & Zuberer, D. A. Soil organic carbon, microbial biomass, and mineralizable carbon and nitrogen in sorghum. *Soil Science Society of America Journal* **59**, 460-466 (1995).
57 Franzluebbers, A. J. & Arshad, M. A. Water-stable aggregation and organic matter in four soils under conventional and zero tillage. *Canadian Journal of Soil Science* **76**, 387-393 (1996).
58 Franzluebbers, A. J., Langdale, G. W. & Schomberg, H. H. Soil carbon, nitrogen, and aggregation in response to type and frequency of tillage. *Soil Science Society of American Journal* **63**, 349-355 (1999).
59 Franzluebbers, A. J. & Stuedemann, J. A. Particulate and non-particulate fractions of soil Organic carbon under pastures in the Southern Piedmont USA. *Environmental Pollution* **116**, 53-62 (2002).
60 Freitas, P. L., Blancaneaux, P., Gavinelly, E., Larre-Larrouy, M. C. & Feller, C. Nivel e natureza do estoque organico de latossols sob diferentes sistemas de uso e manejo. *Pesquisa Agropecuaria Brasilia* **35**, 157-170 (2000).
61 Gál, A., Vyn, T. J., Micheli, E., Kladivko, E. J. & McFee, W. W. Soil carbon and nitrogen accumulation with long-term no-till versus moldboard plowing overestimated with tilled-zone sampling depths. *Soil & Tillage Research* **96**, 42-51 (2007).
62 Garcia-Prechac, F., Ernst, O., Siri-Prieto, G. & Terra, J. A. Integrating no-till into crop-pasture rotations in Uruguay. *Soil & Tillage Research* **77**, 1-13 (2004).
63 Green, V. S., Stott, D. E., Cruz, J. C. & Curi, N. Tillage impacts on soil biological activity and aggregation in a Brazilian Cerrado Oxisol. *Soil & Tillage Research* **92**, 114-121 (2007).
64 Gwenzi, W., Gotosa, J., Chakanetsa, S. & Mutema, Z. Effects of tillage systems on soil organic carbon dynamics, structural stability and crop yields in irrigated wheat (Triticum aestivum L.)-cotton (Gossypium hirsutum L.) rotation in semi-arid Zimbabwe. *Nutrient Cycling in Agroecosystems* **83** (2009).
65 Halvorson, A. D., Vigil, M. F., Peterson, G. A. & Elliott, E. T. *Long-term tillage and crop residue management study at Akron, Colorado*. 361-370 (CRC PRESS, 1997).
66 Halvorson, A. D., Wienhold, B. J. & Black, A. L. Tillage, nitrogen, and cropping system effects on soil carbon sequestration. *Soil Science Society of America Journal* **66**, 906-912 (2002).
67 Havlin, J. L. & Kissel, D. E. *Management effects on soil organic carbon and nitrogen in the east-central Great Plains of Kansas*. 381-386 (CRC Press, 1997).
68 Hernanz, J. L., L opez, R., Navarrete, L. & Sanchez-Giron, V. Long-term effects of tillage systems and rotations on soil structural stability and organic carbon stratification in semiarid central Spain. *Soil & Tillage Research* **66**, 129-141 (2002).

69 Hernanz, J. L., Sanchez-Giron, V. & Navarrete, L. Soil carbon sequestration and stratification in a cereal/leguminous crop rotation with three tillage systems in semiarid conditions. *Agriculture Ecosystems & Environment* **133**, 114-122 (2009).
70 Hertnanz, J. L., Sanchez-Giron, V. & Navarrete, L. Soil carbon sequestration and stratification in a cereal/leguminous crop rotation with three tillage systems in semiarid conditions. *Agriculture Ecosystem & Environment* **133**, 114-122 (2009).
71 Higashi, T. *et al.* Tillage and cover crop species affect soil organic carbon in Andosol, Kanto, Japan. *Soil & Tillage Research* **138**, 64-72 (2014).
72 Hou, R. *et al.* Effects of tillage and residue management on soil organic carbon and total nitrogen in the North China Plain. *Soil Science Society of America Journal* **76**, 230-240 (2011).
73 Hussain, I., Olson, K. R., Wander, M. M. & Karlen, D. L. Adaption of soil quality indices and application to three tillage systems in southern Illinois. *Soil & Tillage Research* **50**, 237-249 (1999).
74 Ismail, I., Blevins, R. L. & Frye, W. W. Long-term no-tillage effects on soil properties and continuous corn yields. *Soil Science Society of America Journal* **58**, 193-198 (1994).
75 Jagadamma, S. & Lal, R. Distribution of organic carbon in physical fractions of soils as affected by agricultural management. *Biology and Fertility of Soils* **46**, 543-554 (2010).
76 Jarecki, M. K. & Lal, R. Crop management for soil carbon sequestration. *Critical Reviews in Plant Sciences* **22**, 471-502 (2010).
77 Jemai, I., Aissa, N. B., Guirat, S. B., Ben-Hammouda, M. & Gallali, T. On-farm assessment of tillage impact on the vertical distribution of soil organic carbon and structural soil properties in a semiarid region in Tunisia. *Journal of Environmental Management* **113**, 488-494 (2012).
78 Jemai, I., Aissa, N. B., S.B., G., Ben-Hammouda, M. & Gallali, T. Impact of three and seven years of no-tillage on the soil water storage, in the plant root zone, under a dry subhumid Tunisian climate. *Soil Tillage & Research* **126**, 26-33 (2013).
79 Karlen, D. L., Kumar, A., Kanwar, R. S., Cambardella, C. A. & Colvin, T. S. Tillage system effects on 15-year carbon-based and simulated N budgets in a tile-drained Iowa field. *Soil & Tillage Research* **48**, 155-165 (1998).
80 Karlen, D. L. *et al.* Long-term tillage effects on soil quality. *Soil & Tillage Research* **32**, 313-327 (1994).
81 Kumar, S., Kadono, A., Lal, R. & Dick, W. Long-term no-till impacts on organic carbon and properties of two contrasting soils and corn yields in Ohio. *Soil Science Society of America Journal* **76**, 1798-1809 (2012).
82 Kumar, S. *et al.* Long-term tillage and drainage influences on soil organic carbon dynamics, aggregate stability and corn yield. *Soil Science and Plant Nutrition* **60**, 108-118 (2014).
83 Lal, R. Soil quality changes under continuous cropping for seventeen seasons of an alfisol in western Nigeria. *Land Degradation and Development* **9**, 259-274 (1998).
84 Lal, R., Mahboubi, A. A. & Fausey, N. R. Long-term tillage and rotation effects on properties of a central Ohio soil. *Soil Science Society of America Journal* **58**, 517-522 (1994).
85 Lammerding, D., Hontoria, C., Tenorio, J. & Walter, I. Mediterranean dryland farming: effect of tillage practices on selected soil properties. *Agronomy Journal* **103**, 382-389 (2010).
86 Larney, F. J., Bremer, E., Janzen, H. H., Johnston, A. M. & Lindwall, C. W. Changes in total, mineralizable and light fraction soil organic matter with cropping and tillage intensities in semiarid southern Alberta, Canada. *Soil & Tillage Research* **42**, 229-240 (1997).
87 Liang, A. *et al.* Short-term effects of tillage practices on soil aggregation fractions in a Chinese Mollisol. *Acta Agriculturau Scandinavica, Section B - Soil and Plant Science* **61**, 535-542 (2011).
88 Liang, A. Z., Zhang, X. P., Fang, H. J., Yang, X. M. & Drury, C. F. Short-term effects of tillage practices on organic carbon in clay loam soil of Northeast China. *Pedosphere* **17**, 619-623 (2007).
89 Liu, E. *et al.* Long-term effects of no-tillage management practice on soil organic carbon and its fractions in the northern China. *Geoderma* **213**, 379-384 (2014).
90 Lopez-Bellido, R., Fontan, J., Lopez-Bellido, F. & Lopez-Bellido, L. Carbon sequestration by tillage, rotations, and nitrogen fertilization in a Mediterranean Vertisol. *Agronomy Journal* **101**, 310-318 (2009).
91 Lopez-Bellido, R. J., Munoz-Romero, V., Fuentes-Guerra, R., Fernandez-Garcia, P. & Lopez-Bellido, L. No-till: A key tool for sequestering C and N in microaggregates on a Mediterranean Vertisol. *Soil & Tillage Research* **166**, 131-137 (2017).

92 Lopez-Fando, C., Dorado, J. & Pardo, M. T. Effects of zone-tillage in rotation with no-tillage on soil properties and crop yields in a semi-arid soil from central Spain. *Soil & Tillage Research* **95**, 226-276 (2007).
93 Lopez-Fando, C. & Pardo, M. T. Changes in soil chemical characteristics with different tillage practices in a semi-arid environment. *Soil & Tillage Research* **104**, 278-284 (2009).
94 Lou, Y., Xu, M., Chen, X., He, X. & Zhao, K. Stratification of soil organic C, N and C:N ratio as affected by conservation tillage in two maize fields of China. *Catena* **95**, 124-130 (2012).
95 Martinez, E., Fuentes, J., Pino, V., Silva, P. & Acevedo, E. Chemical and biological properties as affected by no-tillage and conventional tillage systems in an irrigated Haploxeroll of Central Chile. *Soil & Tillage Research* **126**, 238-245 (2013).
96 Martin-Lammerding, D., Tenorio, J. L., Albarran, M. M., Zambrana, E. & Walter, E. Influence of tillage practices on soil biologically active organic matter content over a growing season under semiarid Mediterranean climate. *Spanish Journal of Agricultural Research* **11** (2013).
97 Mielke, L. N., Doran, J. W. & Richards, K. A. Physical environment near the surface of plowed and no-tilled soils. *Soil & Tillage Research* **7**, 355-366 (1986).
98 Mrabet, R., Saber, N., El-brahli, A., Lahlou, S. & Bessam, F. Total, particulate organic matter and structural stability of a Calcixeroll soil under different wheat rotations and tillage systems in a semiarid area of Morocco. *Soil & Tillage Research* **57**, 225-235 (2001).
99 Murage, E., Voroney, P., Kay, B., Deen, B. & Beyaert, R. Dynamics and turnover of soil organic matter as affected by tillage. *Soil Science Society of America Journal* **71**, 1363-1370 (2006).
100 Nyborg, M., Solberg, E. D., Malhi, S. S. & Izaurralde, R. C. *Fertilizer N, crop residue, and tillage alter soil C and N content in a decade* 93-99 (CRC Press, 1995).
101 Olson, K. R., Lang, J. M. & Ebelhar, S. A. Soil organic carbon changes after 12 years of no-tillage and tillage of Grantsburg soils in Southern Illinois. *Soil & Tillage Research* **81**, 217-225 (2005).
102 Page, K. L. *et al.* Organic carbon stocks in cropping soils of Queensland, Australia, as affected by tillage management, climate, and soil characteristics. *Soil Research* **51**, 596-607 (2013).
103 Pierce, F. J. & Fortin, M. C. *Long-term tillage and periodic plowing of a no-tilled soil in Michigan: Impacts, yield, and soil organic matter*. 141-149 (CRC Press Inc., 1997).
104 Plaza-Bonilla, D., Cantero-Martinez, C. & Alvaro-Fuentes, J. Soil carbon dioxide flux and organic carbon content: effects of tillage and nitrogen fertilization. *Soil Science Society of America Journal* **75**, 1874-1884 (2011).
105 Rhoton, F. E. *et al.* Chemical and physical characteristics of four soil types under conventional and no-tillage systems. *Soil & Tillage Research* **28**, 51-61 (1993).
106 Robertson, F. *et al.* Effect of cropping practices on soil organic carbon: evidence from long-term field experiments in Victoria, Australia. *Soil Research* **53**, 636-646 (2015).
107 Sa, J. C. M. *et al.* Long-term tillage systems impacts on soil C dynamics, soil resilience and agronomic productivity of a Brazilian Oxisol. *Soil & Tillage Research* **136**, 38-50 (2014).
108 Saffigna, P. G., Powlson, D. S., Brookes, P. C. & Thomas, G. A. Influence of sorghum residues and tillage on soil organic matter and soil microbial biomass in an Australian vertisol. *Soil Biology and Biochemistry* **21**, 759-765 (1989).
109 Sainju, U., Caesar, T., Lenssen, A., Evans, R. & Kolberg, R. Tillage and cropping sequence impacts on nitrogen cycling in dryland farming in Eastern Montana, USA. *Soil & Tillage Research* **103**, 332-341 (2009).
110 Sainju, U., Lenssen, A., Caesar-Thonthat, T. & Waddell, J. Carbon sequestration in dryland soils and plant residue as influenced by tillage and crop rotation. *Journal of Environmental Quality* **35**, 1341-1347 (2005).
111 Sainju, U. *et al.* Dryland residue and soil organic matter as influenced by tillage, crop rotation, and cultural practice. *Plant and Soil* **338**, 27-41 (2011).
112 Sainju, U., Singh, B., Whitehead, W. & Wang, S. Carbon supply and storage in tilled and nontilled soils as influenced by cover crop and nitrogen fertilization. *Journal of Environmental Quality* **35**, 1507-1517 (2005).
113 Sainju, U. M., Senwo, Z. N., Nyakatawa, E. Z., Tazisong, I. A. & Reddy, K. C. Soil carbon and nitrogen sequestration as affected by long-term tillage, cropping systems, and nitrogen fertilizer sources. *Agriculture Ecosystem & Environment* **127**, 234-240 (2008).

114 Sainju, U. M., Singh, B. P. & Whitehead, W. F. Long-term effects of tillage, cover crops, and nitrogen fertilization on organic carbon and nitrogen concentrations in sandy loam soils in Georgia, USA. *Soil & Tillage Research* **63**, 167-179 (2002).
115 Salinas-Garcia, J. R., Hons, F. M. & Matocha, J. E. Long-term effects of tillage and fertilization on soil organic matter dynamics. *Soil Science Society of America Journal* **61**, 152-159 (1997).
116 Salinas-Garcia, J. R. *et al.* Tillage effects on microbial biomass and nutrient distribution in soils under rain-fed corn production in central-western Mexico. *Soil & Tillage Research* **66**, 143-152 (2002).
117 Salvo, L., Hernandez, J. & Ernst, O. Distribution of soil organic carbon in different size fractions, under pasture and crop rotations with conventional tillage and no-till systems. *Soil & Tillage Research* **109**, 116-122 (2010).
118 Schomberg, H. & Jones, O. Carbon and nitrogen conservation in dryland tillage and cropping systems. *Soil Science Society of America Journal* **63**, 1359-1366 (1998).
119 Sheehy, J., Six, J., Alakukku, L. & Regina, K. Fluxes of nitrous oxide in tilled and no-tilled boreal arable soils. *Agriculture Ecosystem & Environment* **164**, 190-199 (2013).
120 Shi, X. *et al.* Zone tillage impacts on organic carbon of a clay loam in Southwestern Ontario. *Soil Science Society of America Journal* **75**, 1083-1089 (2011).
121 Shrestha, B. M., Singh, B. R., Forte, C. & Certini, G. Long-term effects of tillage, nutrient application and crop rotation on soil organic matter quality assessed by NMR spectroscopy. *Soil Use and Management* **31**, 358-366 (2015).
122 Sombrero, A. & de Benito, A. Carbon accumulation in soil. Ten-year study of conservation tillage and crop rotation in a semi-arid area of Castile-Leon, Spain. *Soil & Tillage Research* **107**, 64-70 (2010).
123 Sun, B., Hallett, P., Caul, S., Daniell, T. & Hopkins, D. Distribution of soil carbon and microbial biomass in arable soils under different tillage regimes. *Plant and Soil* **338**, 17-25 (2011).
124 Thomas, G. A., Dalal, R. C. & Standley, J. No-till effects on organic matter, pH, cation exchange capacity and nutrient distribution in a Luvisol in the semi-arid subtropics. *Soil & Tillage Research* **94**, 295-304 (2007).
125 Tian, S. *et al.* Continued no-till and subsoiling improved soil organic carbon and soil aggregation levels. *Agronomy Journal* **106**, 212-218 (2013).
126 Tivet , F. *et al.* Soil organic carbon fraction losses upon continuous plow-based tillage and its restoration by diverse biomass-C inputs under no-till in sub-tropical and tropical regions of Brazil. *Geoderma* **209-210**, 214-225 (2013).
127 Ussiri, D. & Lal, R. Long-term tillage effects on soil carbon storage and carbon dioxide emissions in continuous corn cropping system from an alfisol in Ohio. *Soil & Tillage Research* **104**, 39-47 (2009).
128 VandenBygaart, A. J., Yang, X. M., Kay, B. D. & Aspinall, J. D. Variability in carbon sequestration potential in no-till soil landscapes of southern Ontario. *Soil & Tillage Research* **65**, 231-241 (2002).
129 Varvel, G. E. & Wilhelm, W. W. No-tillage increases soil profile carbon and nitrogen under long-term rainfed cropping systems. *Soil & Tillage Research* **114**, 28-36 (2011).
130 Viaud, V., Angers, D. A., Parnaudeau, V., Morvan, T. & Menasseri Aubry, S. Response of organic matter to reduced tillage and animal manure in a temperate loamy soil. *Soil Use and Management* **27**, 84-93 (2010).
131 Wander, M. M., Bidart, M. G. & Aref, S. Tillage impacts on depth distribution of total and particulate organic matter in three Illinois soils. *Soil Science Society of America Journal* **62**, 1704-1711 (1998).
132 Wang, W. J. & Dalal, R. C. Carbon inventory for a cereal cropping system under contrasting tillage, nitrogen fertilization and stubble management practices. *Soil & Tillage Research* **91**, 68-74 (2006).
133 Wright, A. & Hons, F. Soil carbon and nitrogen storage in aggregates from different tillage and crop regimes. *Soil Science Society of America Journal* **69**, 141-147 (2004).
134 Xu, S. Q. *et al.* Soil organic carbon stocks as affected by tillage systems in a double-cropped rice field. *Pedosphere* **23**, 696-704 (2013).
135 Yang, X. M. & Kay, B. D. Impacts of tillage practices on total, loose- and occluded-particulate, and humified organic carbon fractions in soils within a field in southern Ontario. *Canadian Journal of Soil Science* **81**, 149-156 (2001).
136 Yang, X. M. & Wander, M. M. Tillage effects on soil organic carbon distribution and storage in a silt loam soil in Illinois. *Soil & Tillage Research* **52**, 1-9 (1999).

137 Zhang, M., Sparrow, S., Lewis, C. & Knight, C. Soil properties and barley yield under a twenty-years experiment of tillage, straw management and nitrogen application rates in the sub-arctic area of Alaska. *Acta Agriculturea Scandinavica Section B-Soil and Plant Science* **57**, 374-382 (2007).

138 Zhang, X., Xin, X., Zhu, A., Zhang, J. & Yang, W. Effects of tillage and residue managements on organic C accumulation and soil aggregation in a sandy loam soil of the North China plain. *Catena* **156**, 176-183 (2017).
